# Validation of 3D printed MAYO tubes and stethoscope in simulated medical environment – Tools fabricated with additive manufacturing for emergency care

**DOI:** 10.1016/j.heliyon.2023.e20866

**Published:** 2023-10-16

**Authors:** Ferenc Molnar, Matyas Rendeki, Szilard Rendeki, Balint Nagy, Viktor Bacher, Peter Bogar, Adam Schlegl, Arnold Koltai, Peter Maroti, Gergely Marovics

**Affiliations:** a University of Pecs, Medical School, Medical Skills Education and Innovation Centre ,HU-7624, Pecs, Szigeti str. 12, Hungary; b University of Pecs, Clinical Centre, Department of Anesthesiology and Intensive Care HU-7624 ,Pecs, Ifjusag str 13, Hungary; c University of Pecs, Medical School, 3D Printing and Visualization Centre, HU-7624, Boszorkany str. 2, Hungary; d University of Pecs, Clinical Centre, Department of Orthopaedics, HU-7632, Pecs, Akac str. 1, Hungary; e University of Pecs, Medical School, Department of Public Health Medicine ,HU-7624, Pecs, Szigeti str. 12, Hungary

**Keywords:** 3D printing, emergency medicine, MAYO tube, Stethoscope, medical simulation, Resuscitation, ABCDE

## Abstract

Emergency and disaster medical care often face resource or equipment shortages. 3D printing technology has been proven to be effective in cases with insufficient supply chains. MAYO tubes and stethoscopes are essential components of ABCDE patient examinations; however, 3D-printed variants have not been fully tested. These 3D-printed instruments were substituted and validated in a simulated pre-hospital environment.

In total, 26 participants were included in this study. Fifteen clinicians or paramedics with at least 3 years of professional experience and 10 medical students. One student was excluded because he had relevant experience with emergency care. As basic tasks, the placement of MAYO tubes and auscultation with stethoscopes were performed using medical simulators. 3D printed instruments were compared with conventional clinical devices by measuring the time required for the intervention, success rate, and user satisfaction. In the study FFF (Fused Filament Fabrication (FFF), SLS (Selective Laser Sintering (SLS), and SLA (stereolithography) 3D printing were used in this study.

The times required for implementation and auscultation were examined for each instrument. There was no significant difference between the MAYO tube (p = 0.798) and the stethoscope (p = 0.676). In the case of stethoscopy, the study investigated the correct diagnosis, and no significant difference was found (p = 0.239), although an interesting trend was observed. Regarding the MAYO tube, the study found no significant difference in correct position formation (p = 0.163). The experience levels of the groups did not influence these factors. However, significant differences in user satisfaction were found in both cases in favour of the conventional versions (p < 0.001).

Overall, the results of this study suggest that 3D-printed devices could be suitable replacements for clinic-based devices in emergency situations. The 3D-printed devices did not perform inferiorly at any of the indicated points compared to their classical counterparts. However, the practical applicability of the devices used in this study requires further investigation.

## Highlights

• In emergency medical care, the information about 3D-printed instruments is limited.
• Conventional MAYO tubes and stethoscopes compared with 3D printed counterparts.
• FFF, SLS, and SLA 3D printing technology have been involved in the study.
• Time for implementation, success rate, and satisfaction have been measured.
• 3D printed devices have the same effectiveness as standard clinical equipment.

## Graphical abstract

**Figure:**
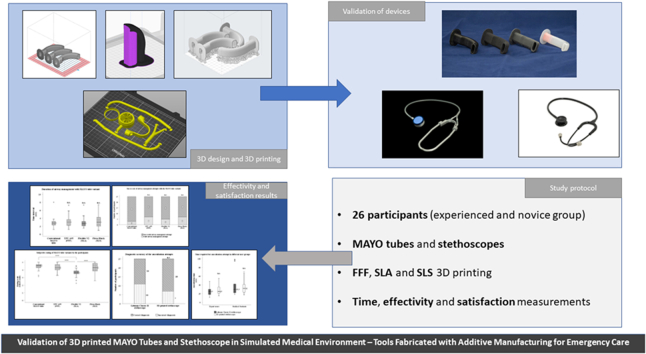

## 1 Introduction

In recent decades, 3D printing technologies have become essential tools in various medical fields [1,2]. Previous studies have shown that it can effectively support the entire medical device development cycle and is considered a fast, cost-effective, and user-friendly method for prototyping [[3], [4], [5]]. In addition, during the COVID-19 pandemic, it played a key role in the production of personal protective equipment (PPE), valves, and adaptors for ventilators, and nasopharyngeal swabs, and teaching models were produced with additive manufacturing [[6], [7], [8], [9]]. Studies have concluded that 3D printing is useful in case of supply shortages, and it is well known that it can help healthcare professionals at remote medical sites or in the case of catastrophic events [[10], [11], [12]].

In the case of critically ill patients who are expected to die without rapid assessment and intervention or to suffer permanent impairment of health [13,14], fast and effective treatment is fundamental regarding patient outcomes and long-term survival. Based on international standards, the ABCDE protocol must be used for critically ill unconscious patients. The first two steps of the procedure (A: airway, B: breathing) are indispensable for maintaining circulation. The airways can be sustained using endotracheal, nasopharyngeal, or MAYO tubes. MAYO tubes are simple airway management devices that are the most common and easiest to use, requiring no special skills or training. They are based on international guidelines and are the first recommended and preferred equipment for definitive treatment [[15], [16], [17]].

After securing the airways, the breathing functions were checked. For this purpose, stethoscopy is the gold standard of medical instruments [18]. These two devices are undoubtedly important for pre-hospital patient management and early treatment of critically ill individuals.

In the case of a catastrophic event, a pandemic, or remote medical sites where supply chains are unavailable or limited, 3D printing can be used to manufacture medical instruments. Previous studies have shown that 3D-printed stethoscopes can be comparable to standard instruments, such as Littmann Cardiology III [19], and smart solutions, such as smartphone-connected stethoscopes, can be fabricated as well [[20], [21], [22], [23]]. A recent study involving four medical students and two instructors examined and proved the usability of 3D-printed stethoscopes in auscultation training, in which they simulated mitral stenosis, mitral regurgitation, and aortic stenosis [24]. Supraglottic airway management devices have been successfully developed and tested on Thiel embalmed bodies [25]; however, no previous work has reported the use of 3D printing for manufacturing MAYO tubes [26].

Although 3D printing technology has been used in almost all fields of medicine and the vast majority of medical instruments have been manufactured and tested in international studies, we did not find previous studies which aimed to critically evaluate 3D printed stethoscopes or MAYO tubes in real or simulated medical environments, involving a significant number of participants. In addition, a recent review indicated that 3D printing in critical care settings is strongly under-researched and under-utilized [26].

This study aimed to validate a 3D-printed stethoscope based on the Glia model [19] in a simulated pre-hospital environment and to prove its diagnostic effectiveness in a larger group of participants with different experience levels in emergency patient care. In addition, as an essential tool in critical care, 3D-printed MAYO tubes were investigated by our research group to determine their applicability and usability in the treatment of critically ill patients.

## 2 Materials and methods

### 2.1 3D design and 3D printing of the medical instruments

For the manufacturing of the stethoscope, 3D models from a previous study were used [19]. The models were processed and repaired using AutoDesk Netfabb [Autodesk Inc.; United States of America, San Francisco]. PrusaSlicer software [[Prusa Research a.s., Czech Republic, Prague] was used for the slicing process. After finalisation of the model, an Original Prusa i3 MK3S + [Prusa Research a.s.; Czech Republic; Prague] desktop FFF (Fused Filament Fabrication) device printed out the parts of the device, using PETG (Polyethylene Terephthalate-G) in a 1.75 mm filament form [Herz Hungária Kft.; Hungary; Üllő]. The infill density was 100 %, and the layer height was set to 200 μm. The stethoscope was printed at a printing speed of 60 mm/s using a 0.4 mm nozzle. The nozzle temperature was set to 240 °C, while the printing bed temperature was 90 °C.

Because there is no previous information about 3D printing of MAYO tubes in the scientific literature, we aimed to test different 3D printing technologies and materials for this purpose. After manual measurements, 3D models were prepared using the Rhinoceros 6 [Robert McNeel & Associates; United States of America; Washington] modelling software, and slicing was performed using printer-specific software. The MAYO tubes were produced using FFF (Ultimaker 3 Extended [Ultimaker B.; Netherlands; Utrecht], slicing: Ultimaker Cura), SLS (Sinterit Lisa rev.C [Sinterit Ltd.; Poland; Kraków], slicing: Sinterit Studio), SLA (stereolithography; Formlabs Form 2 [Formlabs Inc.; United States of America; Massachusetts], slicing: PreForm), 3D printer@@ For the FFF technology, the model was produced using flexible Thermoplastic Polyurethane (TPU) A95[Ultimaker B.; Netherlands; Utrecht]], and Polyvinyl Alcohol (PVA) was used as the support material [Formfutura BV; Netherlands; Nijmegen]. In this case, the nozzle size was 0.4 mm, and the printing speed was set to 25 mm/s. The nozzle temperature was 223 °C and the printing bed temperature was 60 °C. For SLA 3D printing, Flexible V2 [Formlabs Inc.; United States of America; Massachusetts] resin was used, and for SLS, Flexa Black [Sinterit Ltd.; Poland; Kraków] material was selected in powder form. The infill density was 100 % in all cases, and the layer height was 100-100 μm when FFF and SLA printings were carried out, and 125 μm when SLS technology was applied.

### 2.2 Participants

**Fig. 1:**
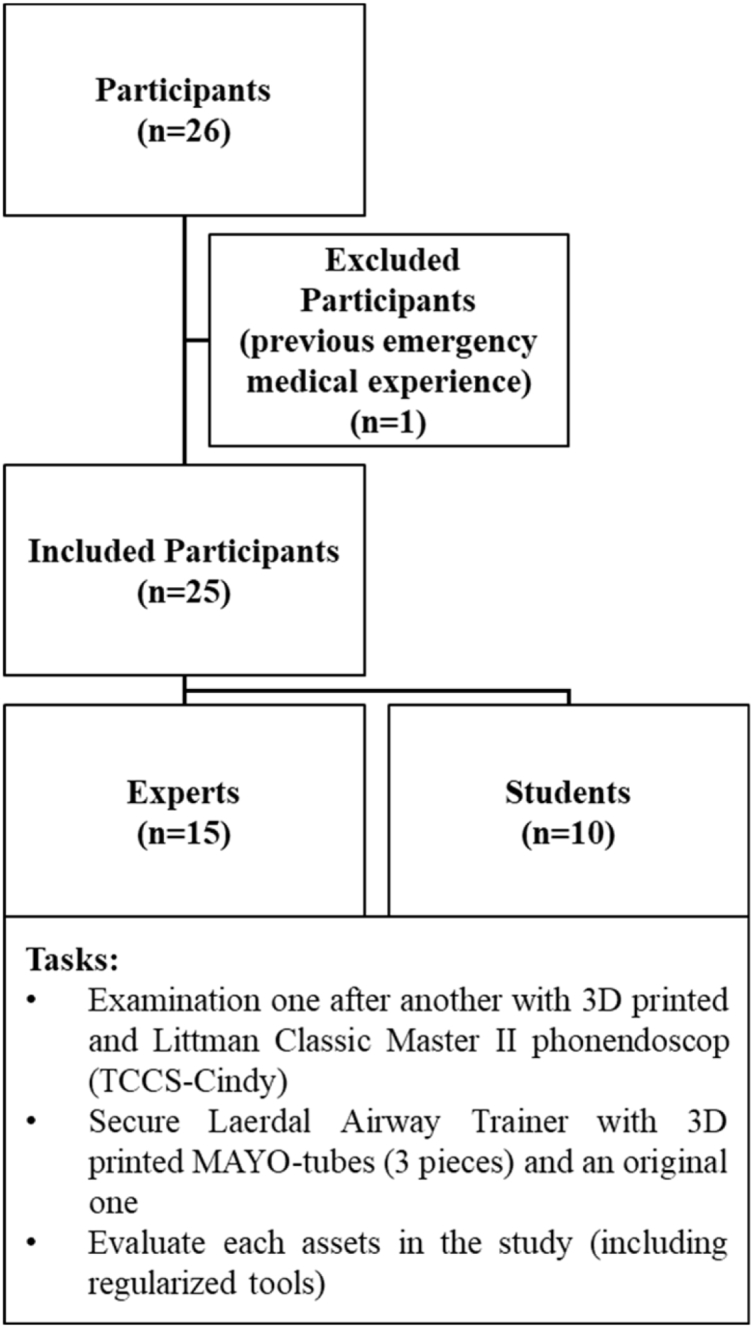
**Flow chart representing the study protocol for participant selection.** A total of 26 participants were initially enrolled for the study. One participant was subsequently excluded from the "medical student" group due to previous emergency medical experience. The remaining 25 participants were then classified into "expert" and "medical student" user groups.

A total of 26 applicants participated in the study. Written consent was obtained from all participants according to the ethical approval of the study (Regional Research Ethical Committee of the University of Pécs Medical School (No. 7176 –PTE 2018). Applicants were divided into two groups based on their knowledge and experience in the fields of catastrophes and emergency medicine. The first group consisted of applicants with advanced clinical experience in this field: resident doctors and specialist doctors from the field of emergency care or anaesthesiology and paramedics with a minimum of 3 years of experience (n^1^ = 16). The second group consisted of medical students from the Medical School at the University of Pécs, Hungary (n^2^ = 10), indicating that these applicants had basic knowledge but no clinical experience in emergency medicine. Medical students were enrolled only in the third year of their medical training after entering the preclinical training phase. One medical student had previous clinical experience due to his part-time job in a pre-hospital; thus, this student was excluded from the study (Fig. 1).

### 2.3 Evaluation criteria for the efficiency of the equipment

**Fig. 2:**
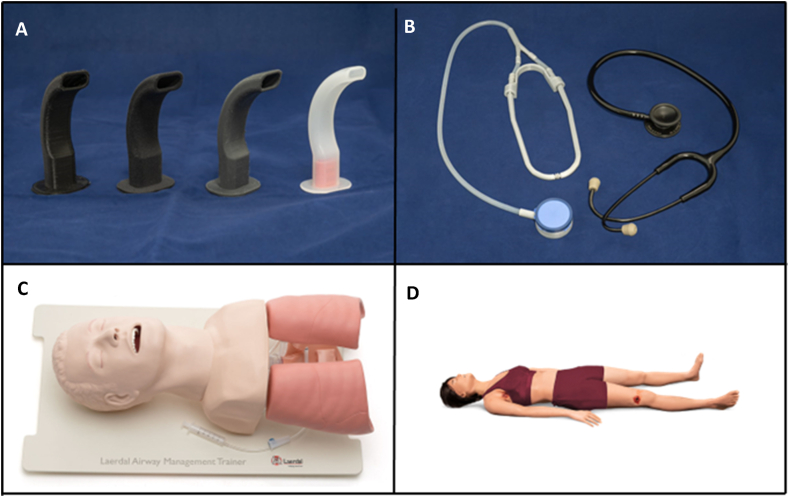
**Instruments and simulators employed in the study.** (A) Sequential images of MAYO tube variants, from left to right: TPU A95, Flexa Black, Flexible V2, and control MAYO tube. (B) Image of a 3D printed stethoscope (grey; left side) alongside a Littman Classic II stethoscope (black; right side). (C) Image of the tactical casualty care simulator "Sindy" utilized in the assessment. (D) Image of the Laerdal® Airway Management Trainer engaged in the evaluation process of the MAYO tube variants. The images have been used by the written consent of the respected owners.

The 3D printed devices were tested in simulated emergency situations, where the applicants had to acquire the required skills while using the instruments on the Laerdal Airway Management Trainer (LATM; USA, 167 Myers Corners Rd, Wappingers Falls, NY, United States, New York)) (Fig. 2.). To measure the efficiency of the stethoscopes, a TCCS-Cindy [TCCS-S] patient simulator system was used (Fig. 2). (USA: 1007 Old Philadelphia RD, Bldg 1 B, Aberdeen, MD 21001). All the used devices and simulators can be seen on Fig. 2(A-D). To evaluate the task completion efficiency of the applicants, standardised evaluation sheets were prepared (Supplementary Material can be found in the Data Repository) for both the MAYO tubes and stethoscopes. The participants completed evaluation sheets after each session. To assess the applicant experience, participants completed a satisfaction survey (Likert Scale) as a feedback evaluation tool [27].

Airway management tasks were performed with all three 3D-printed MAYO tubes and a control clinical instrument. The airway management procedure was considered successful, if, after insertion of the instrument into the LATM, the observer was able to perform 2 successful Bag-Valve-Mask (BVM) ventilation out of 3, meaning that at least 450 ml air volume inflated the artificial lungs of the simulator. The maximum available time for inserting the instrument and three 3 ventilation attempts was 2 min. If the instrument was unable to inflate the artificial lung, at least half of the ventilated air volume inflated the stomach, or the 2-min time limit was exceeded, the attempt failed. This was monitored according to the feedback from the LATM. Each participant attempted airway management using all four instruments and was evaluated. Each participant attempted airway management in a pre-defined order according to the instruments. Similarly, the time interval and success rate were measured to evaluate the stethoscope, and all devices were evaluated by the users using a Likert scale.

In the case of the stethoscope, the participants had to try to detect physiological and pathological lung sounds by examining four pre-defined points on the TCCS-S with both the previously described 3D-printed stethoscope and the control instrument. In this study, the Littman Classic II (3M-Littman - 55424 St. Paul Minnesota, 55144-1000 US) stethoscope served as the control instrument, as it is one of the stethoscopes most commonly used by emergency services and hospitals in Hungary. In each case, the TCCS-S either simulated physiological breathing sounds, or pathologically weaker/absent breathing sounds associated with pneumothorax. For each applicant, physiological and pathological breathing sounds were randomly assigned to one of the stethoscopes. Each applicant then made an auscultation attempt with each stethoscope, and after each attempt, the attendant reported the result of the auscultation to the observer. An auscultation attempt was considered successful if the participant reported the correct breathing sounds to the observer [28,29]. The available time frame was maximised at 3 min for each instrument. If the time limit was exceeded or the participant reported an incorrect auscultation result, the attempt was marked as a failure. In the experiment, the auscultation time (s) and diagnostic accuracy (correct/incorrect diagnosis) were measured along with the level of satisfaction of the participants (Likert scale).

### 2.4 Statistical analysis

SPSS 26.00 (IBM Corporation, Armonk, NY, US) was used for the statistical analysis. The types of statistical tests used are indicated in each figure legend. In each case, the Shapiro-Wilk normality test and Levene's test were used, and the appropriate parametric (ANOVA) or non-parametric (Wilcoxon, Mann-Whitney, or Kruskal-Wallis tests) statistical tests were selected. The chi-square test was used to assess categorical variables.

## 3 Results

### 3.1 Comparison of the 3D printed and the Littmann Classic II stethoscope

**Fig. 3:**
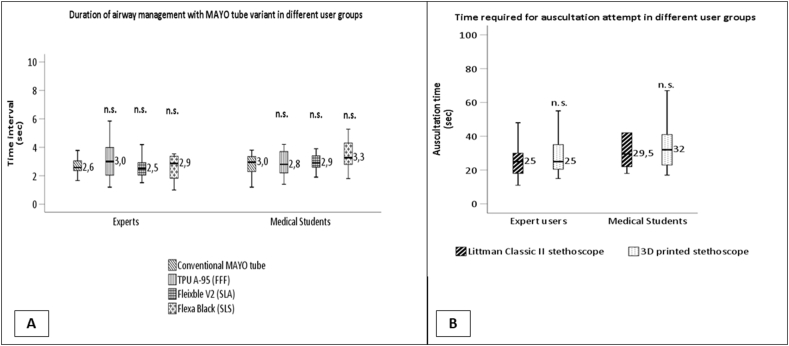
**Comparative analysis of time intervals required for airway management and auscultation tasks between expert and student groups.** (A) Duration of the airway management attempts using MAYO tube variants; n.s.: non-significant (Kruskal-Wallis test, p = 0.798). (B) Comparison of the time interval necessary for the auscultation attempt; n.s.: non-significant (Wilcoxon test, p = 0.864).

**Fig. 4:**
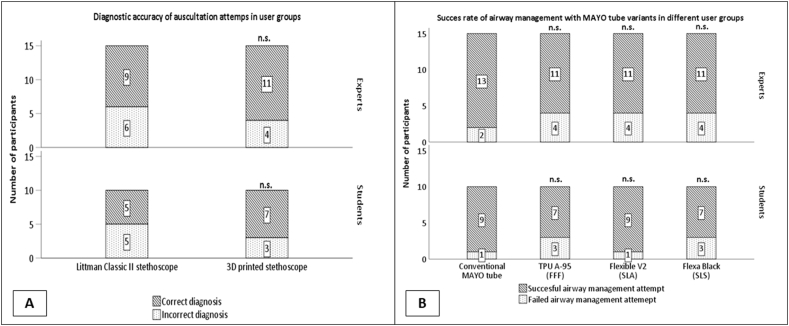
**Success rate and diagnostic accuracy employing all instruments in expert and student groups.** (A) Success rate of airway management attempts with MAYO tube variants; n.s.: non-significant (Chi-square test, p = 0.163). (B) Comparison of the diagnostic accuracy in auscultation attempts; n.s.: non-significant (Chi-square test, p = 0.239).

The stethoscopes were compared based on the participants' auscultation time, diagnostic accuracy, and subjective ratings. The time intervals needed for competition of different tasks are summarized on Fig. 4 (A,B). The auscultation time interval showed no difference between the control and the 3D printed stethoscopes (median ± sd, 29 ± 21.46 s vs. 27 ± 14.43 s, p = 0.864) (Fig. 3B). As previous user experience can greatly influence auscultation time, we also examined the time interval in different user groups. Our results showed no statistically significant differences in auscultation time between user groups (Littmann Classic experts vs. students: p = 0.115, 3D printed experts vs. students: p = 0.216). However, the auscultation time did not differ significantly in either user group (median ± sd, expert user group:25 s ± 10.41 s vs. 25 s ± 10.31 s, p = 0.460; medical student user group:29.5 s ± 29.5 s, 32 s ± 18.36 s, p = 0.552) (Fig. 3.).

Each auscultation attempt resulted in either a correct or incorrect diagnosis; thus, diagnostic accuracy could be determined. Surprisingly, using a 3D printed stethoscope slightly improved the diagnostic accuracy; however, this difference was not significant (ratio of correct/incorrect diagnosis, 14:11 vs. 18:7, p = 0.248) (Fig. 4. A). The Likert scale was used as a multidimensional subjective rating to evaluate the user experience following auscultation attempts. Based on this, the overall rating of the 3D printed stethoscope was significantly lower compared to the Littman classic II (median ± sd, 4.3 ± 0.62 score vs. 3.5 ± 0.59 score, p < 0.001). Despite the 3D-printed stethoscope, the participants made a diagnosis without a significant difference. They rated their ability to auscultate more effectively using the Littman Classic II stethoscope (median ± sd, 5 ± 0.64 score vs. 4 ± 0.9 score, p = 0.004). They also found it to be better suited for this purpose (median ± sd, 5 ± 0.9 score vs. 4 ± 0.9 score, p = 0.005). However, the 3D-printed stethoscope was not affected by the rigidity of plastic. (median ± sd, 5 ± 1.2 score vs. 4 ± 1.2 score, p = 0.098).

### 3.2 Comparison of the 3D printed and classical MAYO tube variants

The different MAYO tubes were compared based on the time interval required for airway management, the success rate of airway management, and subjective ratings by the users. The duration of the airway management attempts showed no significant difference between the different MAYO variants (median ± sd, conventional MAYO tube vs. TPU A95 vs. Flexible V2 vs Flexa Black, 2.79 ± 1.27 s vs. 3 ± 1.49 s vs. 2.7 ± 1.21 s vs. 3 ± 1.76 s, p = 0.798) (Fig. 3. A). Similarly, the success rate of airway management did not differ among different MAYO variants (ratio of successful/failed attempts: conventional MAYO tube vs. TPU A95 vs. Flexible V2 vs. flexible black, 22:3 vs. 18:7 vs. 20:5 vs. 18:7, p = 0.163) (Fig. 4. B). A Likert scale was used to evaluate user experience after airway management attempts.

**Fig. 5:**
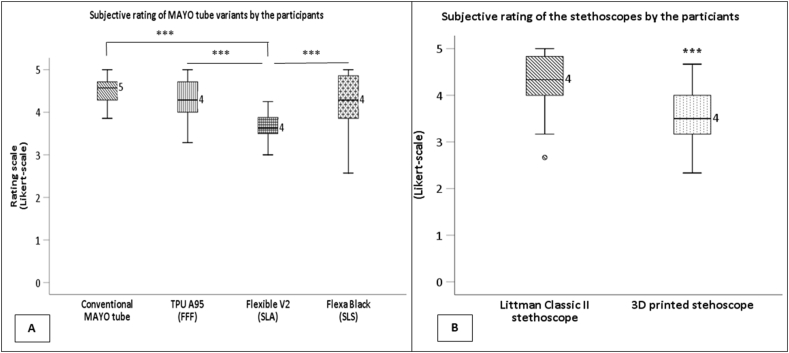
**Participants' subjective ratings of the MAYO tube variants and stethoscopes, using a Likert scale.** (A) MAYO tube variants rating; ***: p < 0.001 (Wilcoxon test, comparisons: conventional MAYO tube vs. Flexible V2 p < 0.001, TPU A95 vs. Flexible V2 p < 0.001, Flexa Black vs. Flexible V2 p < 0.001). (B) Subjective rating of the stethoscopes; ***: p < 0.005 (Wilcoxon test).

The subjective analysis revealed that the Flexible V2 MAYO variant received significantly lower rating scores compared to every other MAYO variant (median ± sd, conventional MAYO tube vs. TPU A95 vs. Flexible V2 vs Flexa Black, 4.57 ± 0.487 score vs. 4.28 ± 0.52 score vs. 3.6 ± 0.54 score vs. 4.28 ± 0.66 score, conventional MAYO tube vs. Flexible V2 p < 0.001, TPU A95 vs. Flexible V2 p < 0.001, Flexa Black vs. Flexible V2 p < 0.001) (Fig. 5). The conventional MAYO tube, TPU A95, and Flexa Black MAYO variants were rated as equally good. Interestingly, the 3D-printed MAYO tubes were copied from the original devices with almost no modifications. The participants found this to be identical to the conventional MAYO tube they were already familiar with (mean ± sd, conventional MAYO tube vs. TPU A95 vs. Flexible V2 vs Flexa Black, 4.48 ± 0.82 score vs. 4.28 ± 0.54 score vs. 4 ± 0.7 score vs. 4.16 ± 0.7 score, p = 0.104). However, they still considered the design to be less good than the conventional MAYO tube (median ± sd, conventional MAYO tube vs. TPU A95 vs. Flexible V2 vs Flexa Black, 5 ± 0.46 score vs. 4 ± 0.51 score vs. 4 ± 0.82 score vs. 4 ± 0.72 score, p < 0,001). The subjective rating of participants are summarized on Fig. 5. (A,B).

## 4 Discussion

The study proved that 3D printing technology can effectively fabricate instruments for emergency medical care in the case of a supply shortage. The usability of 3D-printed medical devices is feasible, regardless of the practitioner's experience. There were no significant differences in diagnostic accuracy or time to implementation. The satisfaction survey showed that participants preferred the tools they learned through practical work regardless of their performance. Previous studies have identified tools that are part of the ABCDE approach to patient assessment [13,18]. Conventional medical devices are more expensive and difficult to access in remote locations or in the case of a catastrophic event; however, they can be produced in a cost-effective form using 3D printing technology of almost the same quality [5,8]. It can also be implemented in a more technologically advanced manner than stethoscopes. 3D printing technology can be connected to electronic devices to digitise the recorded auscultation findings, which can form the basis for further investigation. Further studies have shown that devices made with 3D printing technology can be used for patient testing. Therefore, it was incorporated into the ABCDE approach for rapid emergency and disaster care assessment. 3D-printed devices are state-of-the-art, and their engineering design allows them to be fabricated much more precisely or individually. In certain environments, they can perform better than, or close to, their clinically used counterparts. The present study compared the state-of-the-art, high-standard, high-quality Littman Classic II to a 3D-printed stethoscope. These results were surprising regarding diagnostic accuracy, as no significant differences were observed. However, an interesting trend was observed for the 3D-printed device. The diagnostic accuracy was better in this group. Owing to the low number of participants, repeating the study could yield interesting results if repeated with more participants. The intervention duration was almost the same for both devices. Satisfaction was significantly higher in both groups in favour of conventional clinic-based instruments. The main issue raised by the respondents was the inconvenience of 3D-printed devices.

In addition, different appearances and few similarities between appearance-bothered users. The Littman Classic II scored better in almost all aspects, except for the rigidity of the device, where no significant difference was found. Similar results were obtained for the MAYO tubes. However, no significant differences were found in the correct position or duration of placement. Users clearly preferred conventional MAYO tubes. The duration of the placement and assessment are independent of the type of device and depend more on the clinical experience. This is in line with the previous experience in this study. Experienced providers have better manual skills and are more confident even when newer devices are used.

Technological advances and the growing accessibility of three-dimensional (3D) printing devices have allowed their use in emergency and disaster medicine. However, further investigation is required at this stage, although there was no significant difference in diagnostic accuracy or task execution. Based on the user feedback, it is not suitable for daily use. However, in the case of a disaster where infrastructure and supply chains are damaged, it may be suitable to solve temporary equipment shortages, as presented in previous studies.

The simulated environment and relatively low number of participants limited the study's accuracy. Furthermore, the study did not cost-effectively assess 3D printed devices. Further studies are necessary to regularize 3D printed devices in everyday practice; however, they can be made available to us in a disaster situation. However, repeating the study with more participants forms our future plans, and in an even more complex situational practical environment, expanding the trials to cadavers or real-life situations.

## 5 Conclusion

3D-printed medical devices are considered to possess great potential because of their accessibility, versatility, and ability to customise devices in a wide range of scenarios, including pre-hospital care, remote medical operations, and supply shortages. The presented work examined how additive manufacturing technology can be used to fabricate medical instruments for pre-hospital treatment and how these devices perform compared to their classical counterparts. Involving 25 volunteers and using standard medical simulators, we showed that the efficiency of the 3D printed stethoscope is comparable to that of traditional devices; however, based on subjective user experience, the volunteers preferred the original instruments. MAYO tubes fabricated using different 3D printing technologies (FFF, SLA, and SLS) performed equally well in the evaluations, and their efficacy did not differ from that of commercially available devices. Based on our findings, we conclude that stethoscopes and MAYO tubes can be successfully designed and manufactured using 3D technologies and that these devices show similar performance to their classical counterparts in a simulated medical environment. Our results suggest that 3D-printed devices can replace traditional medical devices in real-life clinical settings. Further trials within simulated or clinical settings with more participants are encouraged.

## Funding information

Project no. TKP2021-NVA-06 has been implemented with the support provided from the National Research, Development and Innovation Fund of Hungary, financed under the TKP2021-NVA funding scheme. The was supported by the project No. 2022–2.1.1-NL-2022-00012 ″National Laboratory of Cooperative Technologies", provided by the Ministry of Culture and Innovation from the National Research, Development and Innovation Fund of Hungary, financed by the National Laboratories program.

## Ethical approval

The study was approved by the Regional Research Ethical Committee of University of Pécs Medical School (No. 7176 –PTE 2018).

## Data availability statement

All the raw data and supplementary material can be found in the Mendeley Data Repository System under the following DOI: *Maroti, Peter; Molnar, Ferenc; Rendeki, Matyas; Rendeki, Szilard; Nagy, Balint; Bacher, Viktor; Bogar, Peter; Schlegl, Adam; Koltai, Arnold (2023), “Data for the publication entitled - " Validation of 3D printed MAYO Tubes and Stethoscope in Simulated Medical Environment – Tools Fabricated with Additive Manufacturing for Emergency Care"”, Mendeley Data, V1*, https://doi.org/10.17632/y4zms5v67t.1; *URL:* *https://data.mendeley.com/datasets/y4zms5v67t/1*.

The 3D model of the stetoschope have been downloaded from the original article of Pavlovsky et al.: *Pavlosky A, Glauche J, Chambers S, Al-Alawi M, Yanev K, Loubani T (2018) Validation of an effective, low cost, Free/open access 3D-printed stethoscope. PLoS ONE 13(3): e0193087.* https://doi.org/10.1371/journal.pone.0193087.

## CRediT authorship contribution statement

**Ferenc Molnar:** Conceptualization, Formal analysis, Investigation, Methodology, Project administration, Writing – original draft, Writing – review & editing. **Matyas Rendeki:** Methodology, Resources, Data curation, Writing – original draft. **Szilard Rendeki:** Resources, Supervision, Validation, Writing – original draft. **Balint Nagy:** Methodology, Resources, Supervision, Validation, Visualization. **Viktor Bacher:** Conceptualization, Investigation, Writing – original draft. **Peter Bogar:** Methodology, Software, Visualization. **Adam Schlegl:** Conceptualization, Investigation, Validation. **Arnold Koltai:** Methodology, Supervision, Validation, Writing – original draft. **Maroti Peter:** Methodology, Project administration, Resources, Supervision, Validation, Writing – original draft, Writing – review & editing. **Gergely Marovics:** Conceptualization, Data curation, Formal analysis, Visualization, Writing – review & editing.

## Declaration of competing interest

The authors declare that they have no known competing financial interests or personal relationships that could have appeared to influence the work reported in this paper.
