# Supplementary material for: Validation of 3D printed MAYO tubes and stethoscope in simulated medical environment – Tools fabricated with additive manufacturing for emergency care
Source: Heliyon. 2023 Oct 16;9(10):e20866. doi: 10.1016/j.heliyon.2023.e20866 (PMC10616327; doi:10.1016/j.heliyon.2023.e20866)
Supplement: Multimedia component 2 [file mmc2.docx]

**Device satisfaction questionnaire**

**Participant code:**


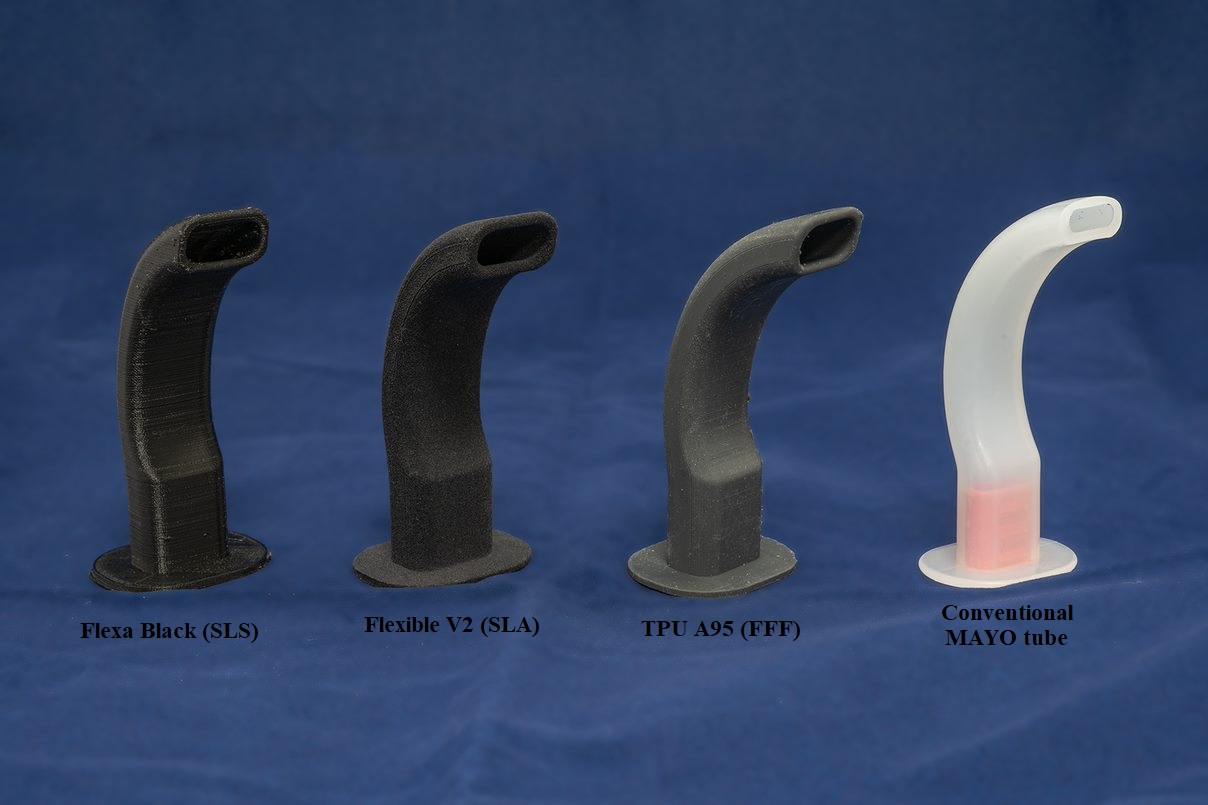


1. How efficiently have you been able to provide airway with **Conventional MAYO tube**?

| 1 - Not at all | 2 | 3 | 4 | 5 - Totally |
| --- | --- | --- | --- | --- |

1. How similar was **Conventional MAYO tube**’s design to the device you used?

| 1 - Not at all | 2 | 3 | 4 | 5 - Totally |
| --- | --- | --- | --- | --- |

1. How similar **Conventional MAYO tube**’s was the way it was inserted to the device you used?

| 1 - Not at all | 2 | 3 | 4 | 5 - Totally |
| --- | --- | --- | --- | --- |

1. 4. How easily were you able to insert the **Conventional MAYO tube** into the model?

| 1 – Very difficult | 2 | 3 | 4 | 5 – Easy unobstructed |
| --- | --- | --- | --- | --- |

1. How would you compare the similarity of the design of the **Conventional MAYO tube** with the device used in the clinic?

| 1 - Not at all | 2 | 3 | 4 | 5 - Totally |
| --- | --- | --- | --- | --- |

1. How did the flexibility of the **Conventional MAYO tube** affect the insertion of the device in the during airway protection?

| 1 – Made it more difficult | 2 | 3 | 4 | 5 – Made it easier |
| --- | --- | --- | --- | --- |

1. How comfortable did you find the **Conventional MAYO tube**?

| 1 – Uncomfortable | 2 | 3 | 4 | 5 – Comfortable |
| --- | --- | --- | --- | --- |

1. Would you use such a **CONVENTIONAL MAYO tube** in your patient care?

**YES/NO**

1. If not, after some modifications, would you consider would you use it?

**YES/NO**

1. How efficiently have you been able to provide airway with **TPU A95 (FFF)**?

| 1 - Not at all | 2 | 3 | 4 | 1. - Totally |
| --- | --- | --- | --- | --- |

1. How similar was **TPU A95 (FFF)**’s design to the device you used?

| 1 - Not at all | 2 | 3 | 4 | 1. - Totally |
| --- | --- | --- | --- | --- |

1. How similar **TPU A95 (FFF)**’s was the way it was inserted to the device you used?

| 1 - Not at all | 2 | 3 | 4 | 1. - Totally |
| --- | --- | --- | --- | --- |

1. How easily were you able to insert the **TPU A95 (FFF)** into the model?

| 1 – Very difficult | 2 | 3 | 4 | 5 – Easy unobstructed |
| --- | --- | --- | --- | --- |

1. How would you compare the similarity of the design of the **TPU A95 (FFF)** with the device used in the clinic?

| 1 - Not at all | 2 | 3 | 4 | 1. - Totally |
| --- | --- | --- | --- | --- |

1. How did the flexibility of the **TPU A95 (FFF)** affect the insertion of the device in the during airway protection?

| 1 – Made it more difficult | 2 | 3 | 4 | 5 – Made it easier |
| --- | --- | --- | --- | --- |

1. How comfortable did you find the **TPU A95 (FFF)**?

| 1 – Uncomfortable | 2 | 3 | 4 | 5 – Comfortable |
| --- | --- | --- | --- | --- |

1. Would you use such a **TPU A95 (FFF)** in your patient care?

**YES/NO**

1. If not, after some modifications, would you consider would you use it?

**YES/NO**

1. How efficiently have you been able to provide airway with **Flexible V2 (SLA)**?

| 1 - Not at all | 2 | 3 | 4 | 5 - Totally |
| --- | --- | --- | --- | --- |

1. How similar was **Flexible V2 (SLA)**’s design to the device you used?

| 1 - Not at all | 2 | 3 | 4 | 5 - Totally |
| --- | --- | --- | --- | --- |

1. How similar **Flexible V2 (SLA)**’s was the way it was inserted to the device you used?

| 1 - Not at all | 2 | 3 | 4 | 5 - Totally |
| --- | --- | --- | --- | --- |

1. How easily were you able to insert the **Flexible V2 (SLA)** into the model?

| 1 – Very difficult | 2 | 3 | 4 | 5 – Easy unobstructed |
| --- | --- | --- | --- | --- |

1. How would you compare the similarity of the design of the **Flexible V2 (SLA)** with the device used in the clinic?

| 1 - Not at all | 2 | 3 | 4 | 5 - Totally |
| --- | --- | --- | --- | --- |

1. How did the flexibility of the **Flexible V2 (SLA)** affect the insertion of the device in the during airway protection?

| 1 – Made it more difficult | 2 | 3 | 4 | 5 – Made it easier |
| --- | --- | --- | --- | --- |

1. How comfortable did you find the **Flexible V2 (SLA)**?

| 1 – Uncomfortable | 2 | 3 | 4 | 5 – Comfortable |
| --- | --- | --- | --- | --- |

1. Would you use such a **Flexible V2 (SLA)** in your patient care?

**YES/NO**

1. If not, after some modifications, would you consider would you use it?

**YES/NO**

1. How efficiently have you been able to provide airway with **Flexa Black (SLS)**?

| 1 - Not at all | 2 | 3 | 4 | 5 - Totally |
| --- | --- | --- | --- | --- |

1. How similar was **Flexa Black (SLS)**’s design to the device you used?

| 1 - Not at all | 2 | 3 | 4 | 5 - Totally |
| --- | --- | --- | --- | --- |

1. 3. How similar **Flexa Black (SLS)**’s was the way it was inserted to the device you used?

| 1 - Not at all | 2 | 3 | 4 | 5 - Totally |
| --- | --- | --- | --- | --- |

1. 4. How easily were you able to insert the **Flexa Black (SLS)** into the model?

| 1 – Very difficult | 2 | 3 | 4 | 5 – Easy unobstructed |
| --- | --- | --- | --- | --- |

1. How would you compare the similarity of the design of the **Flexa Black (SLS)** with the device used in the clinic?

| 1 - Not at all | 2 | 3 | 4 | 5 - Totally |
| --- | --- | --- | --- | --- |

1. How did the flexibility of the **Flexa Black (SLS)** affect the insertion of the device in the during airway protection?

| 1 – Made it more difficult | 2 | 3 | 4 | 5 – Made it easier |
| --- | --- | --- | --- | --- |

1. How comfortable did you find the **Flexa Black (SLS)**?

| 1 – Uncomfortable | 2 | 3 | 4 | 5 – Comfortable |
| --- | --- | --- | --- | --- |

1. Would you use such a **Flexa Black (SLS)** in your patient care?

**YES/NO**

1. If not, after some modifications, would you consider would you use it?

**YES/NO**
