# Supplementary material for: Validation of 3D printed MAYO tubes and stethoscope in simulated medical environment – Tools fabricated with additive manufacturing for emergency care
Source: Heliyon. 2023 Oct 16;9(10):e20866. doi: 10.1016/j.heliyon.2023.e20866 (PMC10616327; doi:10.1016/j.heliyon.2023.e20866)
Supplement: Multimedia component 3 [file mmc3.docx]

**Device satisfaction questionnaire**

**Participant code:**


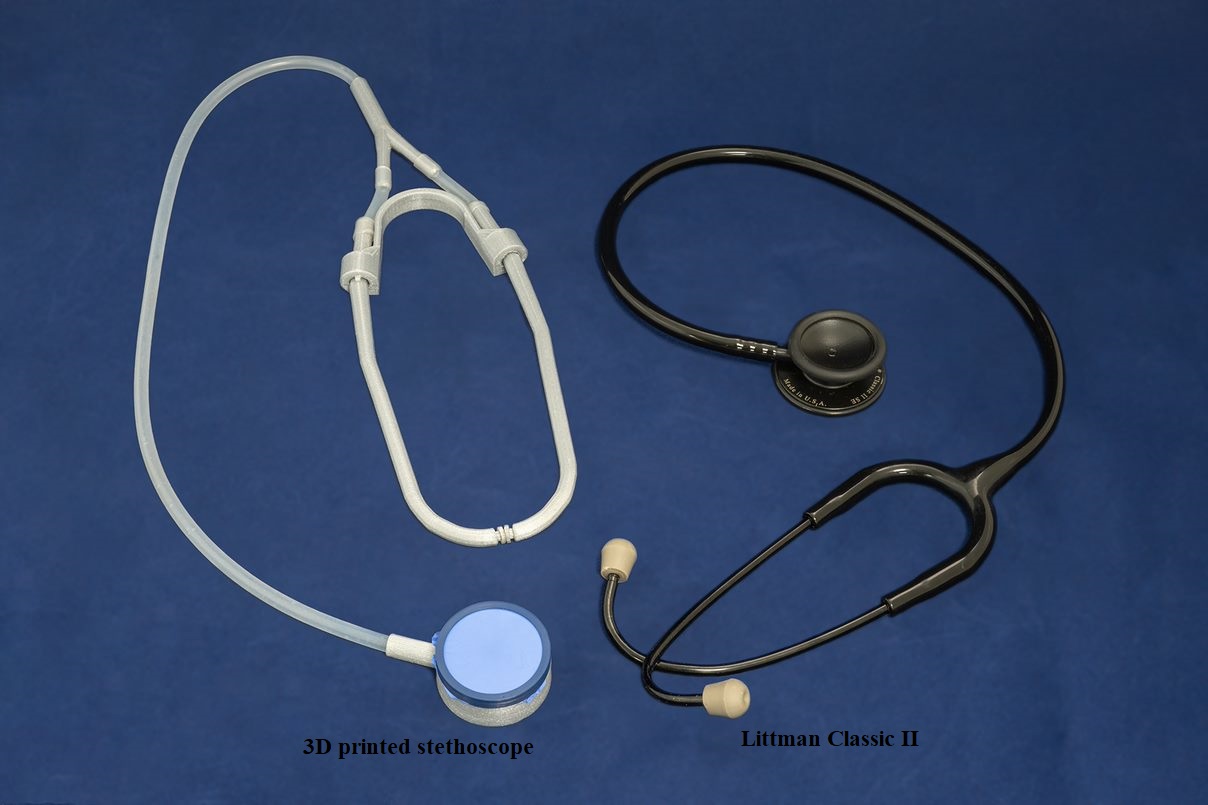


1. How effectively could you auscultate with the **Littman Classic II** stethoscpe?

| 1 - Not at all | 2 | 3 | 4 | 5 - Totally |
| --- | --- | --- | --- | --- |

1. How similar was the design to the **Littman Classic II** stehtoscope you used for this purpose?

| 1 - Not at all | 2 | 3 | 4 | 5 - Totally |
| --- | --- | --- | --- | --- |

1. How similar was **Littman Classic II** stethoscope usability to the device you used?

| 1 - Not at all | 2 | 3 | 4 | 5 - Totally |
| --- | --- | --- | --- | --- |

1. How could you compare the **Littman Classic II** stethoscope similarity of your auscultation in the clinic the device used in the clinic?

| 1 - Not at all | 2 | 3 | 4 | 5 – Totally same |
| --- | --- | --- | --- | --- |

1. How has the hardness of the **Littman Classic II** stethoscope affected the use of the instrument during the assessment?

| 1 - Not at all | 2 | 3 | 4 | 5 - Totally |
| --- | --- | --- | --- | --- |

1. How comfortable did you find the **Littman Classic II** stethoscope?

| 1 – Uncomfortable | 2 | 3 | 4 | 5 – Comfortable |
| --- | --- | --- | --- | --- |

1. Would you use such a **Littman Classic II** in your patient care?

**YES/NO**

1. If not, after some modifications, would you consider would you use it?

**YES/NO**

1. How effectively could you auscultate with the **3D-Printed** stethoscpe?

| 1 - Not at all | 2 | 3 | 4 | 1. - Totally |
| --- | --- | --- | --- | --- |

1. How similar was the design to the **3D-Printed** stehtoscope you used for this purpose?

| 1 - Not at all | 2 | 3 | 4 | 1. - Totally |
| --- | --- | --- | --- | --- |

1. How similar was **3D-Printed** stethoscope usability to the device you used?

| 1 - Not at all | 2 | 3 | 4 | 1. - Totally |
| --- | --- | --- | --- | --- |

1. How could you compare the **3D-Printed** stethoscope similarity of your auscultation in the clinic the device used in the clinic?

| 1 - Not at all | 2 | 3 | 4 | 5 – Totally same |
| --- | --- | --- | --- | --- |

1. How has the hardness of the **3D-Printed** stethoscope affected the use of the instrument during the assessment?

| 1 - Not at all | 2 | 3 | 4 | 5 - Totally |
| --- | --- | --- | --- | --- |

1. How comfortable did you find the **3D-Printed** stethoscope?

| 1 – Uncomfortable | 2 | 3 | 4 | 5 – Comfortable |
| --- | --- | --- | --- | --- |

1. Would you use such a **3D-Printed** in your patient care?

**YES/NO**

1. If not, after some modifications, would you consider would you use it?

**YES/NO**
